# Validation of a high-throughput fermentation system based on online monitoring of biomass and fluorescence in continuously shaken microtiter plates

**DOI:** 10.1186/1475-2859-8-31

**Published:** 2009-06-04

**Authors:** Frank Kensy, Emerson Zang, Christian Faulhammer, Rung-Kai Tan, Jochen Büchs

**Affiliations:** 1 AVT, Biochemical Engineering, RWTH Aachen University, Sammelbau Biologie, Worringerweg 1, 52074 Aachen, Germany; 2 m2p-labs GmbH, Forckenbeckstraße 6, 52074 Aachen, Germany

## Abstract

**Background:**

An advanced version of a recently reported high-throughput fermentation system with online measurement, called BioLector, and its validation is presented. The technology combines high-throughput screening and high-information content by applying online monitoring of scattered light and fluorescence intensities in continuously shaken microtiter plates. Various examples in calibration of the optical measurements, clone and media screening and promoter characterization are given.

**Results:**

Bacterial and yeast biomass concentrations of up to 50 g/L cell dry weight could be linearly correlated to scattered light intensities. In media screening, the BioLector could clearly demonstrate its potential for detecting different biomass and product yields and deducing specific growth rates for quantitatively evaluating media and nutrients. Growth inhibition due to inappropriate buffer conditions could be detected by reduced growth rates and a temporary increase in NADH fluorescence. *GFP *served very well as reporter protein for investigating the promoter regulation under different carbon sources in yeast strains. A clone screening of 90 different *GFP*-expressing *Hansenula polymorpha *clones depicted the broad distribution of growth behavior and an even stronger distribution in *GFP *expression. The importance of mass transfer conditions could be demonstrated by varying filling volumes of an *E. coli *culture in 96 well MTP. The different filling volumes cause a deviation in the culture growth and acidification both monitored via scattered light intensities and the fluorescence of a pH indicator, respectively.

**Conclusion:**

The BioLector technology is a very useful tool to perform quantitative microfermentations under engineered reaction conditions. With this technique, specific yields and rates can be directly deduced from online biomass and product concentrations, which is superior to existing technologies such as microplate readers or optode-based cultivation systems. In particular, applications with strong demand on high-throughput such as clone and media screening and systems biology can benefit from its simple handling, the high quantitative information content and its capacity of automation.

## Background

In order to process large numbers of different clones and to handle the enormous complexity of biological and biochemical systems in modern biotechnology, many research groups have developed new microbioreactor systems. In industry and academic research, the demand for high-throughput and high information content about time-dependent processes has driven the development of microbioreactors. A wide variety of individual solutions for microbioreactors has been suggested and prototyped. Among these, miniature stirred tank reactors [1], gas-inducing impeller systems [2], bubble columns including *in-situ *electrochemical oxygen generation [3,4] and a combination of magnetic stirrer and membrane surface aeration [5] have been developed. Whereas these research groups mainly developed individual solutions for their own laboratories, only a few systems have been commercialized and, therefore, are accessible for a broader research society.

One commercial solution is the SimCell system from Bioprocessors, Inc.. pH, dissolved oxygen tension (DOT) and optical density (OD) values can be recorded in a separate reading station apart from the incubation chamber. This system does not provide sufficient oxygen to microbial cells (specific mass transfer coefficient k_L_a = 10 1/h, [6]) and thus is limited to applications with higher eukaryotic cells such as mammalian cell cultures.

Another miniature bioreactor system (MBR) provided by Microreactor Technologies, Inc. is based on a shaken and gas-sparged 24 well microplate with measurement and control of temperature, pH and DOT. Maximum k_L_a values of 56 1/h have been reported for shaking frequencies of 800 rpm and non-sparged conditions [7].

Whereas new designs of microbioreactors require adapted infrastructure, microtiter plates (MTPs) are already the industrial standard in biotechnology. Therefore, it would be most convenient to maintain this standard and properly adapt operation conditions, geometric design of wells and the measurement method to researcher's needs. Betts et al. [1] have reported that unlike other MBRs, MTPs have the unique advantage, that they provide intrinsic high-throughput capacity and allow automation. MTPs are well characterised in respect to mass transfer. Relatively small k_L_a values of 150–160 1/h are found for standard round 96 well plates [8,9], whereas very high k_L_a values of up to 860 1/h and 1600 1/h are found for square 96 deep well plates and standard round 48 well plates at 200 μL and 300 μL filling volume, respectively [10,11]. These high k_L_a values can only be achieved with very small filling volumes which are often not compatible for further offline analysis or for online analysis in small wells. When working on a small scale, one may likely gain more insight into the micro reactions. Sampling further reduces the filling volume and probably interferes with the reaction. Therefore, online measurements are most favorable.

The most popular and widespread measurement techniques for MTPs are microplate readers. They are equipped with different optical arrangements and generally detect absorbance or fluorescence. Lu et al. [12] performed a study on promoter regulation in *E. coli *with green fluorescent protein (*GFP*) as reporter protein in a standard microplate reader. Common microplate readers have only very poor shaking capacities and no humidity control, simply a temperature control. Therefore, the operation conditions in such readers are also poor with respect to oxygen supply. Inhomogeneous evaporation can lead to misinterpretation of results. Because of the fact, that the measurement procedure in these microplate readers is generally uncoupled from shaking, sedimentation of cells during the measurement process can also cause artifacts. A widespread microplate reader to monitor cell growth is the BioScreen C device [13]. This system features two covered honeycomb plates, each consisting of 100 wells, making it possible to run 200 samples simultaneously. Even through the transparent cover of the plates allows absorption measurements and it reduces the gas exchange with the environment. K_L_a values in the range of that approved for 96 well MTPs (k_L_a = 150–160 1/h, [8]) or even below are supposed, thus limiting its application to anaerobic or low density cultures.

Weiss and John reported about standard 96 well MTPs with immobilized fluorescent dyes on the bottom of the wells. As these so-called optodes are sensitive to pH or DOT, they can only detect one measurement parameter at a time during an experiment [14,15]. These plates can be read with standard microplate readers, yet with the same aforementioned drawbacks. Especially DOT measurements are hindered by shaking interruptions [16]. The same sensors are currently also available in 24 MTPs [17]. To read these optodes, the SensorDish Reader from PreSens can be used [18]. Installed on a shaker, this system can provide real online data without interruption from the shaking process. Here, k_L_a values of up to 250 1/h have been reported with a filling volume of 400 μL [18]. At higher filling volumes (e.g. 1 mL), that are more suitable to optode measurements because the liquid has continuously contact to the optode, k_L_a values are reduced to 100 1/h [18]. These mass transfer conditions can be oxygen-limiting for many fermentations with microbial cells [19,20].

Samorski et al. introduced a new measurement technique which is capable of detecting biomass concentrations via scattered light, NADH, riboflavin or fluorescent proteins through fluorescence in continuously shaken microtiter plates [21]. For the first time, this technique provides real online data about kinetics of biomass and product formation from microbioreactors without any interference of the cultivation.

This paper presents substantial improvements in the online monitoring technique in continuously shaken microtiter plates based on the technique first presented by Samorski et al. [21] and gives several examples of feasible applications. The quantitative detection of biomass concentrations via scattered light for standard microbial expressions systems such as the bacterium *Escherichia coli *and the yeast *Hansenula polymorpha *was studied. Moreover, the influence of different media compositions was evaluated by monitoring of biomass and protein formation while using green fluorescent protein (*GFP*) as a model protein. This technique was further applied to strain and media screening, promoter characterization and evaluation of operation conditions. The application of a soluble fluorescent pH indicator for online detection of pH during fermentation was also studied.

## Methods

### Microorganisms and Media

Standard microbial expression systems, the bacterium *Escherichia coli *and the yeast *Hansenula polymorpha*, were used to evaluate the improved measurement device. The microorganisms were provided by several cooperation partners. The strain *E. coli BL21-Pet 28A ytvAC62A*, expressing a flavin mononucleotide (FMN)-based fluorescent protein (FbFP) [22], was kindly delivered by Thorsten Eggert from evocatal GmbH, Germany (FbFPs reporter proteins are commercialised under the name evoglow^®^, ). The strains *Hansenula polymorpha RB11-pC10-Mox-GFP *and *H.p. RB11-pC10-FMD-GFP *express *GFP *under the control of the *MOX *and the *FMD *promoter, respectively [23]. They were kindly provided by Carsten Amuel from the Institute of Microbiology, Heinrich-Heine-University, Düsseldorf, Germany. For the biomass calibration, wild type strains of *Escherichia coli JM109 *(ATCC 53323) and *Hansenula polymorpha wt *(ATCC 34438, synonym: *Pichia angusta*) were applied.

The *E. coli *experiments were carried out with three different types of bacterial culture media: complex medium Luria Bertani (LB), Terrific Broth (TB) and the synthetic medium for *E. coli *fedbatch fermentations reported by Wilms et al. (WR) [24]. These media had the following compositions: LB medium: 10 g/L tryptone (Difco from Becton Dickinson, USA), 5 g/L yeast extract (Roth, Germany) and 5 g/L NaCl, pH~6.7 without adjustment; TB medium: 5 g/L glycerol (Merck, Germany), 12 g/L tryptone, 24 g/L yeast extract, 12.54 g/L K_2_HPO_4_, 2.31 g/L KH_2_PO_4_, pH~7.2 without adjustment; WR medium: 2.0 g/L Na_2_SO_4_, 2.68 g/L (NH_4_)_2_SO_4_, 0.5 g/L NH_4_Cl, 14.6 g/L K_2_HPO_4_, 4.0 g/L Na_2_HPO_4 _× 2 H_2_O, 1.0 g/L (NH_4_)_2_-H-citrate, 0.5 g/L MgSO_4 _× 7 H_2_O, 0.01 g/L thiamine, 3 ml/L trace element solution (TES), 20 g/L glucose or glycerol, pH was adjusted to 7.2 with 1 M NaOH. TES contains: 0.5 g/L CaCl_2_, 0.18 g/L ZnSO_4 _× 7 H_2_O, 0.1 g/L MnSO_4 _× H_2_O, 10.05 g/L Na_2_-EDTA, 8.35 g/L FeCl_3_, 0.16 g/L CuSO_4_, × 5 H_2_O, and 0.18 g/L CoCl_2 _× 6 H_2_O. The *E. coli *cultures were induced with 0.5 mM isopropyl-β-D-thiogalactopyranosid (IPTG, Biomol, Germany).

To study *H. polymorpha *with different media, the following media were applied: YPG, YPD, YNB-G, YNB-D (buffered and unbuffered). YP medium contained: 20 g/L peptone (Difco from Becton Dickinson, USA), 10 g/L yeast extract (Roth, Germany) and 10 g/L glycerol (YPG) or 20 g/L glucose (YPD), pH~7.6 without adjustment. YNB medium contained: 5 g/L (NH_4_)_2_SO_4 _and 1.7 g/L YNB without ammonium sulfate and amino acids (Difco from Becton Dickinson, USA) and 10 g/L glycerol (YNB-G) or 20 g/L glucose (YNB-D). After all medium components were dissolved, the pH was adjusted to 6.0 with 1 M NaOH. The buffered YNB medium was supplemented with Na_2_HPO4/NaH_2_PO_4 _buffer in a concentration of 0.1 M to maintain the pH at about 6 (starting value pH_0 _= 6.0) during the batch fermentation. In the experiments with different glycerol concentrations, the glycerol concentration in the YNB medium was simply varied during media preparation, whereas the other components remained constant. All the applied chemicals were of analytical grade and were delivered by Fluka (Neu-Ulm, Germany), unless specified otherwise.

### Measurement device

As the measurement device, the same setup like that formerly reported by Samorski et al. [21] was applied with only few but influential changes referring to signal qualities. The major change was varying the distance between the optical light fiber and the microtiter plate bottom as well as the tilting angle. The distance of the optical light fiber to the microtiter plate bottom was reduced from 7 mm to 4 mm, and the tilting angle was increased from 23° to 35°. This adjustment mainly reduced the back scattering of light from internal reflections within the wells, thus stabilizing the measurement signals. Moreover, the flashes of the xenon flash lamp during one measurement were reduced from 200 to 50 flashes to improve the life-time of the lamp. The biomass concentrations were measured via scattered light at 620 nm excitation without an emission filter. The *GFP *concentrations were monitored through an excitation filter of 485 nm and an emission filter of 520 nm. Furthermore, NADH was monitored by an excitation of 340 nm and an emission of 460 nm. The FbFP preferred an excitation of 460 nm and an emission of 520 nm. The sensitivity of the photomultiplier (Gain) was adapted to the different measurement tasks and, therefore, different signal intensities were obtained. The entire device was called "BioLector" in the following text to facilitate referencing of the measurement device. The BioLector holds a data reproducibility of smaller than 5% standard deviation, when cultivating the same clone in the same medium on a microtiter plate. Due to small standard deviation and the high information content, error bars in the figures were omitted.

The pH was measured by adding a sterile solution of HPTS (8-hydroxypyrene-1,3,6-trisulfonic acid trisodium salt, part number: 56360, Fluka, Germany) to TB medium before inoculation with cells. The soluble fluorescent pH indicator was applied in a final concentration of 20 mg/L in the fermentation media. This indicator was excited by filtered xenon light with a wavelength of 410 nm and 460 nm and the emission was detected for both excitation wavelengths at 510 nm. The pH value could be derived from a calibration with buffers in which the same concentration of HPTS (20 mg/L) as in the culture medium was added. Buffers ranging from pH 4.0 to 9.0 and having an ionic strength of 120 mM (20 mM buffer and 100 mM NaCl) were applied to calibrate the measurement device. For each buffer condition, the intensity ratio *I*_*R *_was calculated as follows:

(1)

After determining *I*_*R *_for the different buffers, the pH values were correlated with the Boltzmann equation [25] as follows:

(2)

The calibration parameters pH_O_, dpH, I_R, min _and I_R, max _were calculated with an Excel sheet by using the implied Solver function, determining the least square root of the function (2).

The experiments were exclusively carried out with black standard round 96 well microtiter plates with an optical bottom from Greiner Bio-One, Germany (μclear, part number: 655087), that were covered with a gas permeable membrane from Abgene, UK (part number: AB-0718). If not otherwise specified, the experiments were conducted with 200 μL working volume of culture or medium and normally 995 rpm shaking frequency (shaking diameter of 3 mm). At this operation condition a k_L_a value of 150 1/h was achieved [10].

### Biomass calibration

High concentrations of biomass were required to correlate scattered light intensities and biomass concentrations. To reach high biomass concentrations, the wild type strains of *E. coli *and *H. polymorpha *were cultivated in the complex TB and YPG media in two 250 ml shake flasks over night (20 mL, 37°C, 300 rpm, 50 mm shaking diameter). The cultures were harvested and concentrated ten times by centrifugation (3000 rpm, 5 min, Rotina 38R, Hettich, Germany). One sample (2 mL) of each concentrated cell suspension was taken to determine OD (absorption at 600 nm using a photospectrometer (UVIKON 922, Kontron Instruments, UK)) and cell dry weight (CDW). CDW was determined by washing the cells twice in physiological salt solution (9 g/L NaCl) and drying the cells at 105°C until the mass remained constant. Duplicates of dilutions of the concentrated cell suspension in the respective cultivation medium were then distributed to a fresh microtiter plate and read with the BioLector at the same operating conditions as in the culture experiments. The reference measurement of OD was carried out at 600 nm in a microplate absorption reader (Powerwave X, BioTek, USA) with 200 μL working volume and without any cover.

## Results and discussion

### Biomass calibration

In biotechnology labs, it is very common to follow biomass development by monitoring the optical density. However, it is well known that the correlation between the optical density and biomass concentration is only linear in a very small range – normally between 0.1 to 0.3 OD. For higher biomass concentrations, therefore, it is necessary to adequately dilute the samples and to recalculate the real OD of the sample. It is often mentioned in the literature, that scattered light measurements can compensate for this inconvenience and that biomass concentrations can be correlated up to high densities without dilutions [26]. To prove this, cell suspensions of different biomass concentrations (CDW) of *Hansenula polymorpha wt *in YPG medium were compared by scattered light and optical density measurements.

**Figure 1:**
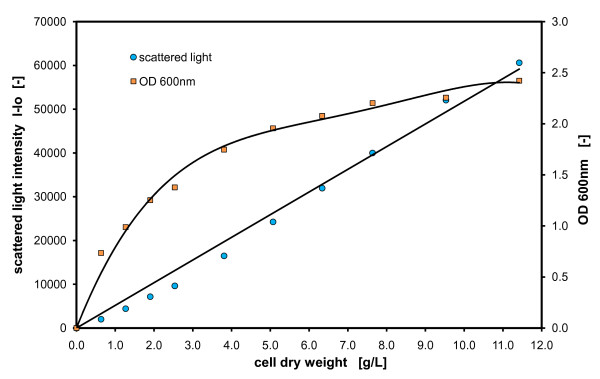
**Comparison between scattered light and optical density measurements (absorption)**. Hansenula polymorpha (wt) culture in YPG medium measured in 96 well MTP, with 200 μL filling volume, at 37°C temperature, 995 rpm shaking frequency and 3 mm shaking diameter, scattered light (ex: 620 nm/em: -, Gain: 40).

Figure 1 shows the correlation of measurement signals from OD and scattered light versus the cell dry weight concentrations of *H. polymorpha*. The OD signals follow a typical saturation curve and confirm that linearity is limited already at concentrations well below 1 g/L CDW. However, the scattered light signals show a good linear correlation with the cell dry weight concentration versus the applied concentrations of up to 11 g/L CDW, which is a normal concentration reached in batch fermentations [2]. Additionally, it is observable that the resolution of the biomass signal with scattered light is very high. Thus, each g/L cell dry weight can be resolved with 5500 scattered light units at the applied sensitivity (Gain = 40). As the OD measurement loses resolution with higher biomass concentration, it is not recommendable to use this method with undiluted samples. Thus, this method is not applicable for online monitoring of cultures.

There are two further aspects which make OD measurements impractical for online monitoring of growing cultures in microtiter plates. First of all, gas-permeable membranes are normally used to seal the wells, to avoid contaminations and to reduce evaporation. This makes transmission measurements almost impossible. Second, the OD measurements are normally conducted when the shaking of the plate is interrupted. This results in measurement artifacts through the sedimentation of cells during the measurement. Furthermore, the decrease in oxygen mass transfer and effectiveness of mixing during the shaking interruption has to be taken into account with sensitive microorganisms or when studying fast metabolic reactions.

### Limits of biomass detection with scattered light

**Figure 2:**
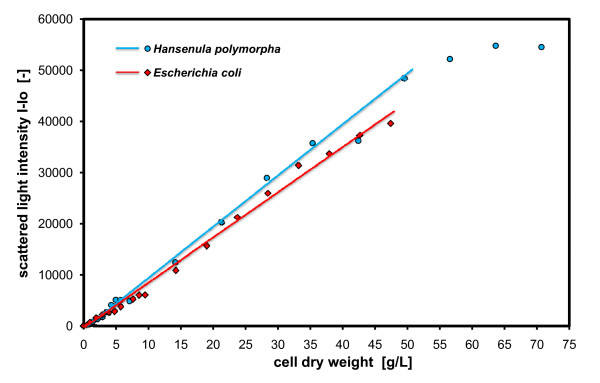
**Limits of biomass monitoring with scattered light**. *Hansenula polymorpha (wt) *in YPG medium; *E. coli JM109 *in TB medium, both were measured in 96 well MTP, with 200 μL filling volume, at 37°C temperature, 995 rpm shaking frequency and 3 mm shaking diameter, scattered light (ex: 620 nm/em: -, Gain: 5).

To determine the limits of the scattered light measurements, a concentrated biomass solution of *E. coli JM109 *and *H. polymorpha wt *after growth in shake flasks was diluted, distributed on a microtiter plate and measured with the BioLector. The results are presented in Figure 2.

In Figure 2 the scattered light intensities of the dilutions series is shown versus the cell dry weight of the cell suspension. It is notable that the scattered light signals show a good linear correlation with the cell dry weight concentrations for both cell types, *E. coli *and *H. polymorpha*, over a broad concentration range. Up to cell concentrations of 50 g/L cell dry weight, the scattered light intensities show a linear correlation with the cell dry weight concentrations. At cell concentrations higher than 50 g/L cell dry weight, the scattered light signal for *H. polymorpha *becomes saturated, for the described measurement setup. At this point, resolution dramatically decreases and further monitoring of undiluted samples is not recommendable. The cell suspension of *E. coli *was followed only up to cell dry weight concentrations of 50 g/L, because the biomass yield of the applied shake flask cultivations was limited to this value. Nevertheless, it is assumed that the detection limit of *E. coli *cell suspensions lies also in the range of 50 g/L cell dry weight, because up to this concentration the signals for yeast cells behave similarly to those of bacteria. However, this conclusion cannot be generalized and extended to any other cell type, media composition or application. To ensure reliable measurement data, it is recommendable to perform a specific biomass calibration when working with new cell types and media. In general, the broad linear range and sensitivity of non-invasive scattered light measurements reflecting biomass concentrations, for the first time, enable online monitoring of cell growth in microbioreactors. Thereby, dilutions of samples, contaminations due to open vessels and interruption of shaking is avoided. If 50 g/L cell dry weight is accepted as upper detection limit for a linear range calibration of scattered light measurements, there is obviously no limitation of biomass detection for batch fermentations. This most commonly applied operation mode in screening [27] reaches a cell dry weight of usually below 15 g/L [2]. The minimal detection limit for biomass concentration ranges from 0.1 to 0.2 g/L CDW (OD600 ~0.2 to 0.6) for bacteria and yeast cells (data not shown here). When flash lamps are used as excitation light (such as in our example), the flashed light can cause oscillations of the scattered light signals at the beginning of the cultivation with normally small cell concentrations as well as in non-inoculated media. This results from interferences of the flashed excitation light and the continuously changing path length of the shaken liquid inside the wells. At small cell densities or in pure media, the light can completely penetrate the liquid and scatter back from the adhesive sealing membrane covering the microtiter plate. With higher cell densities, the light cannot completely penetrate through the liquid anymore, and the path length of the liquid has a reduced effect on signal stability.

### Variation of the amount of carbon source

**Figure 3:**
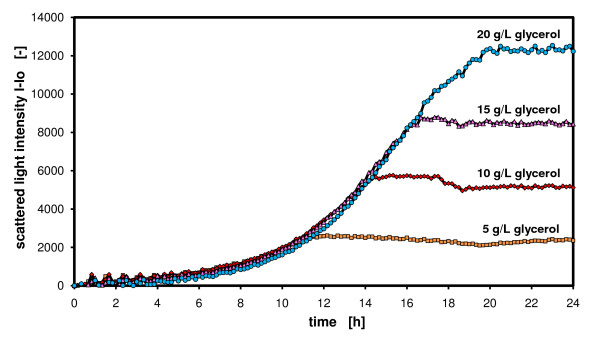
**Comparison of different media – variation of carbon source concentration monitored with scattered light intensities**. *Hansenula polymorpha RB11-pC10-FMD-GFP *culture in YNB-G medium with varying glycerol concentrations (5, 10, 15 and 20 g/L); measured in 96 well MTP, with 200 μL filling volume, at 30°C temperature, 995 rpm shaking frequency and 3 mm shaking diameter, scattered light (ex: 620 nm/em: -, Gain: 10).

Another possibility to calibrate or reference the scattered light signals to biomass concentrations is to grow cells on different amounts of carbon source, assuming a constant biomass yield on the carbon source and no by-product formation. To analyze this, YNB medium with four different concentrations of glycerol (5, 10, 15 and 20 g/L) was prepared and used in a *H. polymorpha *fermentation in microtiter plates. Stöckmann et al. [28] reported that *H. polymorpha *strains do not produce by-products during cultivation on glycerol even under oxygen-limited culture conditions. Figure 3 presents the results of this experiment. A parallel growth of *H. polymorpha *in the exponential growth phase on all four media is observed. Obviously, there is no substrate inhibition on growth. Remarkably, the culture attains the stationary phase at different times. The scattered light levels depend on the available amount of carbon source (glycerol). The more glycerol is present proportionally more biomass can be produced, which is well depicted by the scattered light signals.

### Influence of pH conditions on growth

A large number of different culture media has to be tested to optimize the environment for production strains and to ensure maximum productivity. Unfortunately, pH conditions are often not considered. The most simple way to handle this aspect in small scale cultures is to use buffers.

**Figure 4:**
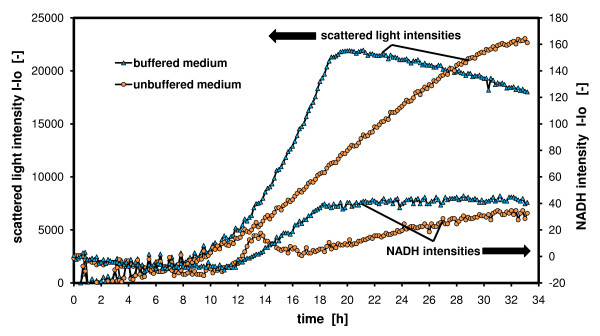
**Influence of pH conditions on growth – buffered/unbuffered medium monitored with scattered light intensities and NADH fluorescence intensities**. *Hansenula polymorpha RB11-pC10-FMD-GFP *culture in buffered YNB medium with 10 g/L glycerol and 100 mM phosphate and unbuffered YNB medium with 10 g/L glycerol without phosphate; measured in 96 well MTP, with 200 μL filling volume, at 30°C temperature, 995 rpm shaking frequency and 3 mm shaking diameter, scattered light (ex: 620 nm/em: -, Gain: 20), NADH (ex: 340 nm/em: 460 nm, Gain: 20).

Figure 4 depicts the effect of buffers on growth of *H. polymorpha*. Here, the cell growth in buffered and unbuffered YNB media is presented. The biogenic NADH pool is simultaneously monitored in both media. The scattered light of the growing yeast cells on buffered medium follows the typical exponential growth curve until entering the stationary phase at 19 hours. The NADH signal also follows an exponential growth curve after a slight decay of the signal at the beginning. In the stationary phase, the NADH signals stay constant, while the scattered light signals decrease slightly and continuously due to probable morphological changes of the cells. The cells in the unbuffered medium, however, behave differently. There seems to be no difference in the monitored signals up to 12 hours of the culturing time. But then, due to a pH decrease below 4.0 (data not shown here), the cells change their metabolism, thereby reflecting the higher proton gradient in the cell environment. This also leads to a decreased growth rate. This point is also reflected by a spontaneous increase in the NADH signal. Probably the respiratory chain is blocked and the NADH pool fills up. After a metabolic switch, the cells grow at a reduced growth rate, and the NADH pool is continuously decreased until 17 hours when it reached the normal level of intracellular NADH (see as reference NADH of the culture in the buffered medium). From this point on, the NADH signal continuously increases again in parallel to the growing cell mass. Due to the unsuitable pH conditions, the yeast can only grow linearly, not exponentially which leads to a much larger cultivation time (Δt = +14 h). Here, there is no evidence that other by-products are formed, because the biomass still reaches the same scattered light levels. By applying other microorganisms or media, the behavior can completely differ with respect to by-product formation, product formation and biomass development. Especially sensitive microorganisms such as e.g. *E. coli *can stop growth when passing below a critical pH~5.0 [20].

### Comparison of different media with *Hansenula polymorpha*

It is also interesting to compare biomass yield, growth rate, protein expression on different mineral and complex media. Therefore, the yeast *H. polymorpha *was grown on different standard expression media for small scale cultures and the growth behavior was monitored with the BioLector.

**Figure 5:**
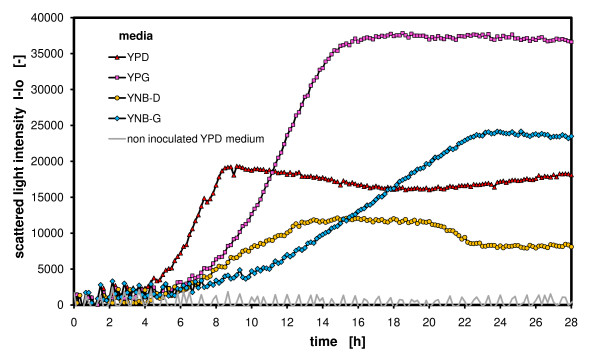
**Comparison of different media – *Hansenula polymorpha *on complex and synthetic media with glucose and glycerol as carbon source monitored with scattered light intensities**. *Hansenula polymorpha RB11-pC10-Mox-GFP *culture in YPG, YPD, buffered YNB-D and YNB-G media; measured in 96 well MTP, with 200 μL filling volume, at 37°C temperature, 995 rpm shaking frequency and 3 mm shaking diameter, scattered light (ex: 620 nm/em: -, Gain: 20).

Figure 5 depicts the differences in growth of the yeast *H. polymorpha *on YPD, YPG, YNB-D and YNB-G medium. The YP medium consists of complex ingredients such as yeast extract and peptones, whereas the YNB medium consists only of a pure mineral basis. The biomass development is clearly graduated with respect to the biomass yield as follows: YPG > YNB-G > YPD > YNB-D and with respect to the specific growth rate as follows: YPD > YPG > YNB-D > YNB-G. It can be clearly stated for both media compositions that glucose (D) is consumed by the cells significantly faster than glycerol (G) and that the biomass yield on glycerol is approximately double that on glucose (at normalized molar mass, as applied here). On the same carbon source, the complex media gained higher biomass yields than the synthetic media resulting from the additional carbon sources supplied by the yeast extract and peptones. Also, this beneficially contributes to the growth rates, because many catabolites are immediately available in the medium and do not have to be synthesized by the cells as in case of the mineral medium YNB.

### Comparison of different media with *Escherichia coli*

**Figure 6:**
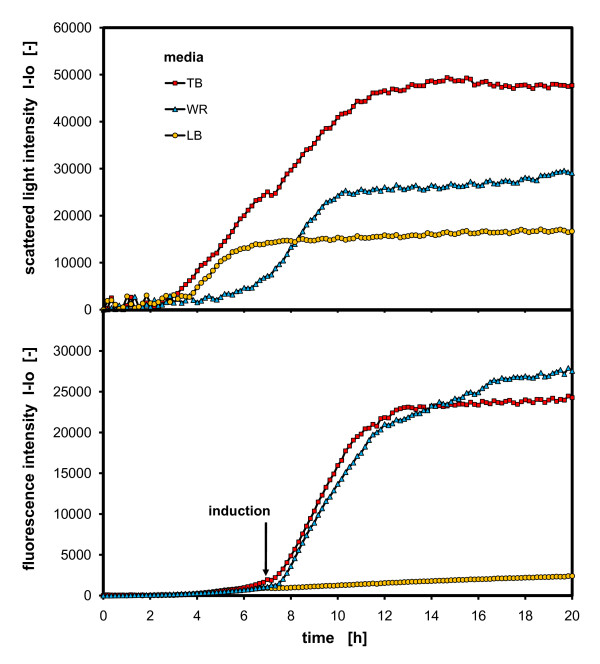
**Comparison of different media – growth and protein expression of a flavin mononucleotide (FMN)-based fluorescent protein (*FbFP*) in *E. coli***. *E. coli BL21-Pet 28A ytvAC62A *culture in LB, TB and WR media; measured in 96 well MTP, with 200 μL filling volume, at 30°C temperature, 995 rpm shaking frequency and 3 mm shaking diameter, induction at 7.3 h with 0.5 mM IPTG, scattered light (ex: 620 nm/em: -, Gain: 20), *FbFP *(ex: 460 nm/em: 520 nm, Gain: 10).

To prove the applicability of the BioLector in media screening, a media comparison was also performed with *E. coli*. The growth and the expression of FbFP (fluorescence protein) were studied on standard media for *E. coli*: LB, TB and WR, the latter being a synthetic fedbatch fermentation medium. Figure 6 presents the results of this experiment.

Interestingly, a clear difference in the biomass development can be seen in this experiment. *E. coli *grows to the highest biomass concentration on TB medium, whereas half the biomass concentration is attained on WR medium and 1/3 of the maximum concentration on LB. While a typical exponential growth curve is observable on the WR medium, the cultures on LB and TB medium show only a linear slope. Moreover, a shift in growth on the TB medium is displayed at 7 h. This has already been described previously by Losen et al. and can be related to a carbon source shift from glycerol to peptone [20]. This shift is also expressed in a turnaround of pH from a decrease to a pH increase at this point [20]. The cultures can grow much faster on complex media (LB and TB) than on the synthetic media due to the availability of key intermediates and building blocks for growth in the complex media. Concerning to the protein expression in this media, it is notable that the expression of the *FbFP *in TB and WR medium gained almost the same level, although the biomass level in TB medium is significantly higher. Even though the expression of the *FbFP *in LB medium is very low, this can also be attributed to the late induction time for the LB culture. At the induction time (7.3 h), the culture already enters the stationary growth phase and no carbon source is left to produce the protein. Prior to induction in all media, there is only a small fluorescence intensity observable which is explained by biogenic fluorescence of the cells or a small constitutive expression of *FbFP*. After the induction, the promoters are activated and fluorescence of the *FbFP *increases strongly in TB and WR medium.

Distinct differences in biomass concentrations and growth rates can be seen using varying carbon source quantities, media backgrounds and buffer conditions. With these results, the applicability of the BioLector technology is confirmed for quantitative biomass and fluorescent protein monitoring as well as for media screening.

### Clone Screening

The BioLector was then applied in clone screening with *H. polymorpha*, expressing *GFP *as a model protein for simplifying quantification. The clones were passaged 8 times for integration of the *GFP*-gen into the *Hansenula *genome and then stabilized [30]. The screening was performed with glycerol as sole carbon source; that means that the applied *FMD *and *MOX *promoters were derepressed and, therefore, they actively expressed *GFP*.

**Figure 7:**
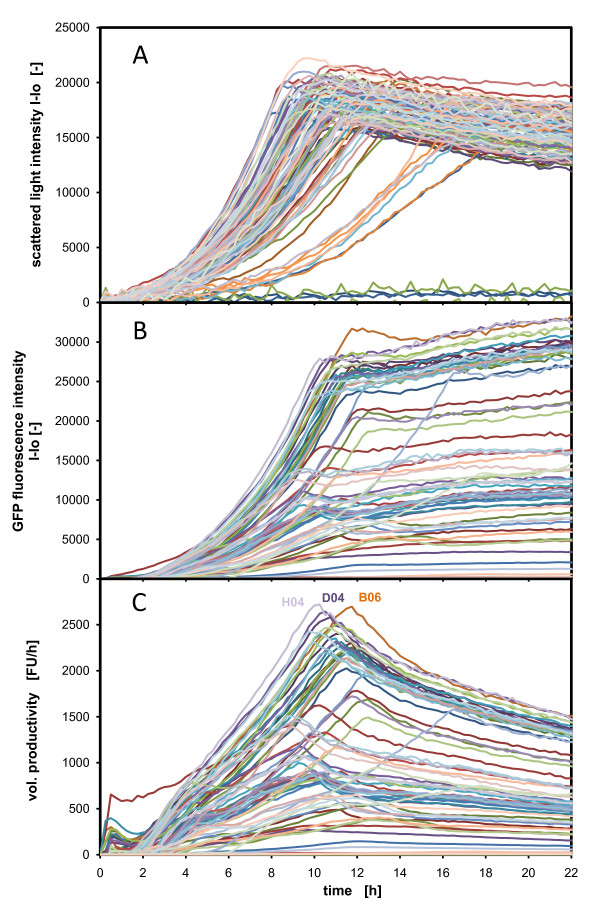
**Clone Screening – comparison of growth and *GFP *protein expression of 90 different *Hansenula polymorpha *clones**. (**A**) growth via scattered light intensities (**B**) protein expression via *GFP *fluorescence intensities; (**C**) volumetric productivity (P_V_) – calculated as *GFP *formation rate without consideration of setup time of the equipment, the best clones are depicted by given the well number in the diagram; 45 clones of *Hansenula polymorpha RB11-pC10-Mox-GFP *and 45 clones of *Hansenula polymorpha RB11-pC10-FMD-GFP *in buffered YNB-G medium; measured in 96 well MTP, with 200 μL filling volume, at 37°C temperature, 995 rpm shaking frequency and 3 mm shaking diameter, scattered light (ex: 620 nm/em: -, Gain: 20), *GFP *(ex: 485 nm/em: 520 nm, Gain: 10).

Figure 7 illustrates the results from a screening of 90 different clones. The scattered light and the fluorescence of *GFP *were monitored during the batch fermentation in a 96 well microtiter plate. Most of the clones grow exponentially, and the majority of clones enter the stationary phase between 8 and 13 h. About seven clones show a significantly reduced growth rate and enter the stationary phase after 15 h and more. Two strains do not grow and form a baseline together with the two wells of non inoculated media. Notably, the different clones achieve different biomass yields, which is expressed in the different scattered light intensities. As the measurement device has a standard deviation of smaller than 5%, the deviation of +/- 15% around the median of the scattered light signals in the stationary phase demonstrates that the biomass yields differ significantly. Figure 7B illustrates the huge diversity of *GFP *formation kinetics and the *GFP *expression levels ranging from a few thousand fluorescent units (FUs) up to 30.000 FUs. It has to be assumed that this huge difference in protein level is also observed in the case of the expression of real target proteins instead of *GFP*. The *GFP *expression kinetics in Figure 7B show a similar exponential curve like the biomass curves in Figure 7A but with a much broader distribution. As formerly reported, the *GFP *protein is very stable [31]. That is proven here by the constant *GFP *fluorescence in the stationary phase. Other proteins probably show degradation and very often are exposed to proteolysis [32,33]. In this case, online monitoring of biomass development and protein formation kinetics can provide valuable information in early bioprocess development. Generating this extensive amount of online information necessitates further data analysis. One simple example of analysis is applying typical evaluation criteria such as the specific product yield (*Y*_*P*/*X*_) which expresses the relation of the formed product to biomass.

**Figure 8:**
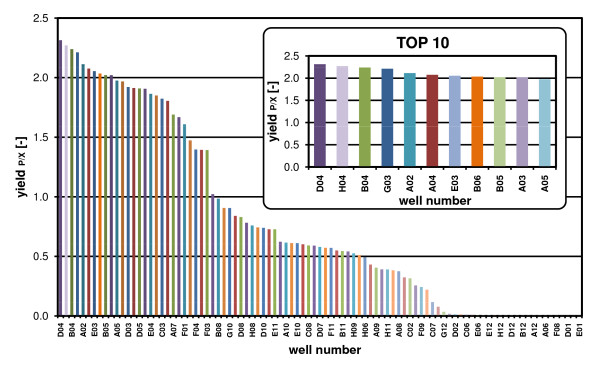
**Derivation of specific product yield (Y_P/X_)**. Data taken from the clone screening of Fig. 7, specific product yield Y_P/X _calculated as ratio of *GFP *intensities (protein concentration) to scattered light intensities (biomass concentration); data arranged to present the best clones from left to right given the well number on the abscissa, TOP10 represents the best ten clones in respect to Y_P/X_.

Figure 8 reveals the calculated specific product yield at 18 h of all *H. polymorpha *clones described (criteria: all clones entered the stationary phase). The whole bar chart of Figure 8 depicts all clones, whereas the zoomed bar chart presents the ten best clones (TOP10). The bar chart discloses the huge diversity of the specific product yield and, therefore, the necessity of sophisticated screening.

Another evaluation criteria of a bioprocess could be the volumetric productivity (P_V_) of the process. Figure 7C visualizes the development of the volumetric productivity in all 96 wells of the microtiter plate. The volumetric productivity is derived from the *GFP *fluorescence units (FU equivalent to product concentrations ~ mg/L) divided by the cultivation time (net productivity, without setup time). Again, very diversified curves are observable. All volumetric productivities reach a peak where the cultures should be harvested. The well numbers of the best clones are highlighted in the diagram of Figure 7C. With respect to the volumetric productivity, it would be most advisable to continue the bioprocess development with these clones. The point of maximum volumetric productivity reflects the expected process performance and the optimum harvest point. Again, the online measured *GFP *fluorescence can be regarded as a convenient model in this case, but alternatives such as fusion proteins with fluorescent reporter proteins or offline protein analysis could be a more realistic case in respect to the industrial praxis. Thus, a sophisticated evaluation of clones and fermentation conditions can already be performed with the hereby presented tools in a very early stage in bioprocess development.

### Characterization of promoters

Promoter regulation is another very important aspect when an expression system or a bioprocess is evaluated. It is still difficult to generate meaningful data on promoter regulation. Currently many laborious techniques are applied, e.g. fluorescence microscopy or fluorescence activated cell sorting (FACS). It is possible to obtain kinetics of the promoter activity or the protein expression through painstaking sampling from culture vessels. Very often promoter activity is controlled by addition of specific inductors or by growth on specific substrates.

**Figure 9:**
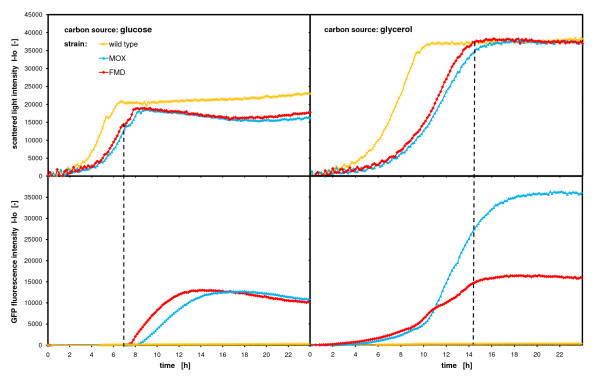
**Characterization of promoters**. MOX and FMD promoter regulation in *Hansenula polymorpha *on glucose and glycerol growth medium monitored via *GFP *fluorescence intensities and parallel measurement of scattered light intensities; *Hansenula polymorpha wt, RB11-pC10-Mox-GFP *and *RB11-pC10-FMD-GFP *in YPD (10 g/L glucose) and YPG (20 g/L glycerol) medium; measured in 96 well MTP, with 200 μL filling volume, at 37°C temperature, 995 rpm shaking frequency and 3 mm shaking diameter, scattered light (ex: 620 nm/em: -, Gain: 20), *GFP *(ex: 485 nm/em: 520 nm, Gain: 10).

Figure 9 presents one example of promoter regulation with *H. polymorpha*. Two different promoters, *MOX *and *FMD*, were studied together with the wild type strain as negative control. Both promoters were inserted in front of the *GFP *gene in the transformation plasmid to follow promoter activity by the fluorescence of *GFP *[23]. The yeast strains were cultivated on two different carbon sources to study the promoter regulation on glucose and glycerol. Figure 9 clearly depicts the differences in growth and promoter activity of the strains on the different media. On both carbon sources, the sequence of growth is equal, whereas the wild type strain grows faster than the recombinant strains. In addition, these two recombinant strains grow at a similar growth rate, with the *FMD *strain growing slightly faster. Proving the findings in Figure 5, the growth rates of all strains are higher on glucose than on glycerol. The biomass yield on glycerol seems to be twice as high as on glucose (on the same molar mass). On glucose, there seems to be a second growth phase following a slight decrease in the scattered light signals at 5 h for the wild type strain and at ca. 7 h for the *MOX *and *FMD *strains. In this latter phase, the cells metabolize the overflow metabolites, ethanol and acetic acid, formed during the growth on glucose [28,29]. The lower diagram of Figure 9 shows the *GFP *formation of the strains. On glucose, there seems to be no promoter activity at all in the presence of glucose (first growth phase). Just after the switch to the second growth phase (on ethanol and acetic acid), the promoters are activated and *GFP *is formed. The *FMD *and *MOX *strains form *GFP *at similar concentrations and in the same growth sequence. Probably also complex components of the medium (YP basis), such as peptone and yeast extract, is utilized to form *GFP*, as no other substrate is available and *GFP *is still formed in the stationary phase. As expected, the wild type does not form any *GFP*. On glycerol however, the promoters behave completely different. Already during the growth phase and in the presence of elevated glycerol concentrations, the promoters are activated and *GFP *is produced. The activity of both promoters is very similar up to 10–11 hours. After that point, the *MOX *promoter activity is boosted while the *FMD *promoter declines. After entering the stationary phase, both promoter activities slow down and the *GFP *fluorescences remain constant. At the end of the fermentation, the *GFP *fluorescence intensity of the *MOX *promoter is about 2.5 times higher than that of the *FMD *promoter, demonstrating the superiority of the *MOX *promoter under the applied experimental conditions. Again, the wild type does not form any *GFP*. This scheme of promoter regulation for *H. polymorpha *is also reported in the literature, where on glucose, *MOX *and *FMD *promoter are repressed by the substrate, whereas on glycerol, these promoters are derepressed at relatively high substrate concentrations [28,29,34,35]. With the help of the BioLector, it is possible to quantitatively characterize promoter activities under different regulation conditions. At the same time, these promoter activities can be assigned to different growth phases and substrate uptakes.

### Influence of filling volume

**Figure 10:**
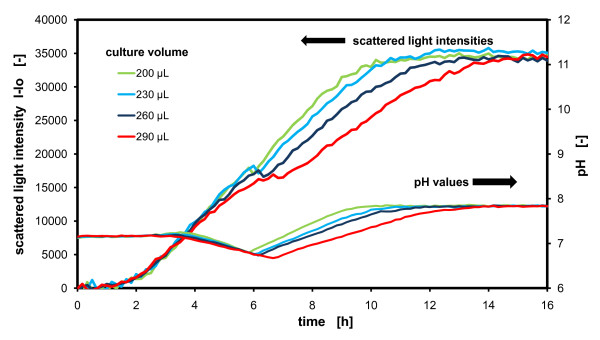
**Influence of filling volume**. Growth and pH monitoring of *E. coli *cultures with filling volumes from 200 μl to 290 μL; *E. coli BL21 *culture in TB medium, measured in 96 well MTP, with 200, 230, 260 and 290 μL filling volume, at 30°C temperature, 995 rpm shaking frequency and 3 mm shaking diameter, scattered light (ex: 620 nm/em: -, Gain: 20), HPTS (ex: 410 nm and 460 nm/em: 510 nm, Gain: 10), calibration parameters for the Boltzmann equation: *I*_*R*, *min *_= 0.00, *I*_*R*, *max *_= 3.00, *dpH *= 0.50, *pH*_0 _= 7.20.

The operation conditions in small-scale cultivations should be characterized and generally be transferable to larger scales. Upon working with microbial expressions systems, one of the most relevant engineering parameters is the oxygen transfer rate. By simply varying the culture volume, the oxygen transfer rate is changed. A good example of the influence of filling volume and, therefore, the oxygen transfer rate in small scale cultures is given by Figure 10.

In Figure 10 growth and pH of an *E. coli *culture are monitored in the complex TB medium which is known to be a strong oxygen-demanding medium (OTR~ 0.07 mol/L/h, [20]). Different culture volumes of 200 μl, 230 μL, 260 μL and 290 μL are applied in a standard 96 well MTP. With higher culture volume, the growth rate is reduced and the fermentation time is prolonged due to a reduced oxygen transfer rate. Additionally, the pH decay is more pronounced in the cultures with higher volumes, because under limited oxygen transfer conditions, the bacteria are forced to produce organic acids from glycerol. More acids are produced under higher oxygen limitation. The diauxic shift of the culture from glycerol to peptones, reflected by the slight decay of the scattered light signals in the middle of the growth curves, also correlates very well with the pH shift from a descending to ascending trend. At first glance, the different mass transfer conditions seem to have no influence on the final biomass yield and the final pH; but, of course, different growth rates and acidifications are observed. It has to be taken into account that even inferior mass transfer conditions and media with lower buffer capacity, than those applied here, could also lead to adverse effects such as termination of biomass growth or instability of the produced protein of interest. In conclusion, undefined culture conditions can result in a unattended selection pressure, the wrong design of a process and finally can cause unwanted problems during scale up.

The soluble fluorescent pH dye (HPTS) applied here could be a good alternative to normally expensive pH optodes. It is easy to apply in any kind of microtiter plate and can be read out with normal fluorescence plate readers. The main drawback of this method is the intensive calibration which has to be performed by the researcher itself and on any new reader. In contrast, pH optodes are normally delivered with calibration parameters.

## Conclusion

The presented microtiter plate based fermentation system can be used to quantify microbial biomass concentrations of up to cell dry weights of 50 g/L without any dilution and in a linear correlation with offline biomass values. This allows the reliable online monitoring of biomass development during fermentation without the need of sampling and dilutions. The parallel monitoring of fluorescent protein concentrations (e.g. *GFP *or derivatives) or other fluorescent analytes in the culture broth allows, for the first time, the online calculation of typical evaluation criteria of a bioprocess such as yield, specific growth and product formation rates as well as volumetric productivity. Of course, online product data are not available in every product screening, however, continuous development in molecular biology and biomarker research will eventually create broader applications of fluorescent reporter proteins in the future [36]. A currently applied solution is fusing *GFP *or any other fluorescent protein to the target protein [37]. This provides a fluorescent signal of the fusion protein and the possibility for online detection. After expression of the fusion protein, the fluorescent protein partner can be cleaved from the target protein during the downstream processing by specific proteases [38]. Another more extensive method to evaluate the product yield is to combine offline analyzed data with online data. Therefore, manual or automated sampling out of the microtiter plate would be helpful. To reduce the sampling frequency and, therefore, the volume removed during sampling, the online biomass signal can be used for triggering the sampling. A good sampling point could be the middle of the exponential growth phase, the entry into the stationary phase and the late stationary phase. Combining these methods of online monitoring and offline protein and nutrient analysis can deliver more sophisticated information and evaluation criteria for bioprocesses. Another interesting metabolite, NADH, can also be monitored with the BioLector technology. Under aerobic and unlimited growth conditions, the NADH fluorescence can be correlated with the biomass concentration, whereas under oxygen, pH or nutrient limitations, the signal can be a valuable indicator of limitations or metabolic changes [39]. Furthermore, the technique can be utilized to evaluate different media by comparing such parameters as biomass concentration, specific growth rate, yield and product formation rate. Even diauxic growth shifts can be detected upon using media with different carbon sources. Thus, this quantitative method can positively reverse media development from merely being empirical experimentation to quantitative and knowledge-based media design. The combination of the BioLector technology with modern genetic engineering enables the real-time characterization of genetic elements such as promoters combined with fluorescent proteins directly during the cultivation. A huge variety of different substrates, chemical compounds and fermentation parameters can be easily studied via the fluorescence of the reporter protein. One aspect which is very often underrated in small-scale fermentations is the effect of engineering parameters. Upon performing high-throughput experimentation on a small scale, it would be most attractive, if the experimental results can be directly transferred to larger reactor scales. Therefore, small-scale reactors such as microtiter plates should be well characterized regarding relevant engineering parameters. OTR_max _and k_L_a values for common microtiter plates of the 48 and 96 well type have already been characterized [8,11]. In respect of validation of the data from micro-scale fermentations with lab fermenter data further work is necessary. This scale up issue will be addressed by the authors of this paper in a separate paper in the near future. In conclusion, the BioLector technology is a powerful tool to generate more insight in bioprocesses in a high-throughput and quantitative manner. It is very suitable for clone screening, media optimization, systems biology and early bioprocess development. Future research in this area should advance this technology towards the integration of optodes for monitoring pH, DOT and other fermentation parameters. The proof of scalability to stirred tank fermenters and the extension to other cell types such as plant, mammalian and human cell lines are highly desirable.

## Competing interests

The authors declare that they have no competing interests.

## Authors' contributions

FK carried out most of the experiments and wrote the manuscript. EZ performed the comparison between scattered light and optical density measurements. CF carried out the *E. coli *cultivations with different filling volumes and online pH detection. RKT contributed the biomass dilutions to determine the limit of linear correlation between scattered light intensities and cell dry weight. FK and JB conceived the study, and participated in its design, coordination, and drafting of the manuscript. All authors read and approved the final manuscript.

## References

[B1] Betts JI, Baganz F (2006). Miniature bioreactors: current practices and future opportunities. Microb Cell Fact.

[B2] Puskeiler R, Kaufmann K, Weuster-Botz D (2005). Development, parallelization, and automation of a gas-inducing milliliter-scale bioreactor for high-throughput bioprocess design (HTBD). Biotechnol Bioeng.

[B3] Doig SD, Ortiz-Ochoa K, Ward JM, Baganz F (2005). Characterization of oxygen transfer in miniature and lab-scale bubble column bioreactors and comparison of microbial growth performance based on constant k(L)a. Biotechnol Prog.

[B4] Maharbiz MM, Holtz WJ, Howe RT, Keasling JD (2004). Microbioreactor arrays with parametric control for high-throughput experimentation. Biotechnol Bioeng.

[B5] Zanzotto A, Szita N, Boccazzi P, Lessard P, Sinskey AJ, Jensen KF (2004). Membrane-aerated microbioreactor for high-throughput bioprocessing. Biotechnol Bioeng.

[B6] The Simcell Technology. http://www.bioprocessors.com.

[B7] Isett K, George H, Herber W, Amanullah A (2007). Twenty-four-well plate miniature bioreactor high-throughput system: assessment for microbial cultivations. Biotechnol Bioeng.

[B8] Hermann R, Lehmann M, Büchs J (2003). Characterization of gas-liquid mass transfer phenomena in microtiter plates. Biotechnology and Bioengineering.

[B9] Ortiz-Ochoa K, Doig SD, Ward JM, Baganz F (2005). A novel method for the measurement of oxygen mass transfer rates in small-scale vessels. Biochemical Engineering Journal.

[B10] Hermann R, Walther N, Maier U, Büchs J (2001). Optical method for the determination of the oxygen-transfer capacity of small bioreactors based on sulfite oxidation. Biotechnology and Bioengineering.

[B11] Kensy F, Zimmermann HF, Knabben I, Anderlei T, Trauthwein H, Dingerdissen U, Buchs J (2005). Oxygen transfer phenomena in 48-well microtiter plates: determination by optical monitoring of sulfite oxidation and verification by real-time measurement during microbial growth. Biotechnol Bioeng.

[B12] Lu C, Bentley WE, Rao G (2004). A high-throughput approach to promoter study using green fluorescent protein. Biotechnol Prog.

[B13] BioScreen C. http://www.bioscreen.fi.

[B14] Weiss S, John GT, Klimant I, Heinzle E (2002). Modeling of mixing in 96-well microplates observed with fluorescence indicators. Biotechnology Progress.

[B15] John GT, Klimant I, Wittmann C, Heinzle E (2003). Integrated optical sensing of dissolved oxygen in microtiter plates: A novel tool for microbial cultivation. Biotechnology and Bioengineering.

[B16] Wittmann C, Kim HM, John G, Heinzle E (2003). Characterization and application of an optical sensor for quantification of dissolved O2 in shake-flasks. Biotechnol Lett.

[B17] Kensy F, John GT, Hofmann B, Büchs J (2005). Characterisation of operation conditions and online monitoring of physiological culture parameters in shaken 24-well microtiter plates. Bioprocess Biosyst Eng.

[B18] PreSens. http://www.presens.de.

[B19] Anderlei T, Zang W, Büchs J (2004). Online respiration activity measurement (OTR, CTR, RQ) in shake flasks. Biochem Eng J.

[B20] Losen M, Frohlich B, Pohl M, Büchs J (2004). Effect of oxygen limitation and medium composition on Escherichia coli fermentation in shake-flask cultures. Biotechnology Progress.

[B21] Samorski M, Muller-Newen G, Büchs J (2005). Quasi-continuous combined scattered light and fluorescence measurements: a novel measurement technique for shaken microtiter plates. Biotechnol Bioeng.

[B22] Drepper T, Eggert T, Circolone F, Heck A, Krauss U, Guterl JK, Wendorff M, Losi A, Gartner W, Jaeger KE (2007). Reporter proteins for in vivo fluorescence without oxygen. Nat Biotechnol.

[B23] Amuel C, Gellissen G, Hollenberg CP, Suckow M (2000). Analysis of Heat Shock Promoters in Hansenula polymorpha: The TPS1 Promoter, a Novel Element for Heterologous Gene Expression. Biotechnol Bioprocess Eng.

[B24] Wilms B, Hauck A, Reuss M, Syldatk C, Mattes R, Siemann M, Altenbuchner J (2001). High-cell-density fermentation for production of L-N-carbamoylase using an expression system based on the Escherichia coli rhaBAD promoter. Biotechnol Bioeng.

[B25] Schulte A, Lorenzen I, Bottcher M, Plieth C (2006). A novel fluorescent pH probe for expression in plants. Plant Methods.

[B26] Junker BH, Wang DIC, Hatton TA (1988). Fluorescence Sensing of Fermentation Parameters Using Fiber Optics. Biotechnol Bioeng.

[B27] Buchs J (2001). Introduction to advantages and problems of shaken cultures. Biochem Eng J.

[B28] Stockmann C, Maier U, Anderlei T, Knocke C, Gellissen G, Buchs J (2003). The oxygen transfer rate as key parameter for the characterization of Hansenula polymorpha screening cultures. J Ind Microbiol Biotechnol.

[B29] Jeude M, Dittrich B, Niederschulte H, Anderlei T, Knocke C, Klee D, Buchs J (2006). Fed-batch mode in shake flasks by slow-release technique. Biotechnol Bioeng.

[B30] Stockmann C, Losen M, Dahlems U, Knocke C, Gellissen G, Buchs J (2003). Effect of oxygen supply on passaging, stabilising and screening of recombinant Hansenula polymorpha production strains in test tube cultures. FEMS Yeast Res.

[B31] March JC, Rao G, Bentley WE (2003). Biotechnological applications of green fluorescent protein. Appl Microbiol Biotechnol.

[B32] Weydemann U, Keup P, Piontek M, Strasser AWM, Schweden J, Gellissen G, Janowicz ZA (1995). High-level secretion of hirudin by Hansenula polymorpha – authentic processing of three different preprohirudins. Appl Microbiol Biotechnol.

[B33] Viaplana E, Rebordosa X, Pi-ol J, Villaverde A (1997). Secretion-dependent proteolysis of recombinant proteins is associated with inhibition of cell growth in Escherichia coli. Biotechnology Letters.

[B34] Hartner FS, Glieder A (2006). Regulation of methanol utilisation pathway genes in yeasts. Microb Cell Fact.

[B35] Oh KS OK, Oh YW, Sohn MJ, Soongee J, Kim YK, Min-Gon K, Rhee SK, Gellissen G, Kang HA (2004). Fabrication of a partial genome microarray of the methylotrophic yeast Hansenula polymorpha: Optimization and evaluation of transcript profiling. J Microbiol Biotechnol.

[B36] Su WW (2005). Fluorescent proteins as tools to aid protein production. Microb Cell Fact.

[B37] Li J, Bentley W, Rao G (2001). Secretion of GFP and GFP-fusion proteins in Saccharomyces cerevisiae. Abstracts of papers of the American Chemical Society.

[B38] Zhang A, Gonzalez SM, Cantor EJ, Chong S (2001). Construction of a mini-intein fusion system to allow both direct monitoring of soluble protein expression and rapid purification of target proteins. Gene.

[B39] Marose S, Lindemann C, Scheper T (1998). Two-dimensional fluorescence spectroscopy: a new tool for online bioprocess monitoring. Biotechnol Prog.

